# Aggregation of Human S100A8 and S100A9 Amyloidogenic Proteins Perturbs Proteostasis in a Yeast Model

**DOI:** 10.1371/journal.pone.0058218

**Published:** 2013-03-06

**Authors:** Ekaterina Eremenko, Anat Ben-Zvi, Ludmilla A. Morozova-Roche, Dina Raveh

**Affiliations:** 1 Department of Life Sciences, Ben-Gurion University of the Negev, Beer-Sheva, Israel; 2 National Institute for Biotechnology in the Negev, Ben-Gurion University of the Negev, Beer-Sheva, Israel; 3 Department of Medical Biochemistry and Biophysics, Umeå University, Umeå, Sweden; UMCG, The Netherlands

## Abstract

Amyloid aggregates of the calcium-binding EF-hand proteins, S100A8 and S100A9, have been found in the corpora amylacea of patients with prostate cancer and may play a role in carcinogenesis. Here we present a novel model system using the yeast *Saccharomyces cerevisiae* to study human S100A8 and S100A9 aggregation and toxicity. We found that S100A8, S100A9 and S100A8/9 cotransfomants form SDS-resistant non-toxic aggregates in yeast cells. Using fluorescently tagged proteins, we showed that S100A8 and S100A9 accumulate in foci. After prolonged induction, S100A8 foci localized to the cell vacuole, whereas the S100A9 foci remained in the cytoplasm when present alone, but entered the vacuole in cotransformants. Biochemical analysis of the proteins indicated that S100A8 and S100A9 alone or coexpressed together form amyloid-like aggregates in yeast. Expression of S100A8 and S100A9 in wild type yeast did not affect cell viability, but these proteins were toxic when expressed on a background of unrelated metastable temperature-sensitive mutant proteins, Cdc53-1p, Cdc34-2p, Srp1-31p and Sec27-1p. This finding suggests that the expression and aggregation of S100A8 and S100A9 may limit the capacity of the cellular proteostasis machinery. To test this hypothesis, we screened a set of chaperone deletion mutants and found that reducing the levels of the heat-shock proteins Hsp104p and Hsp70p was sufficient to induce S100A8 and S100A9 toxicity. This result indicates that the chaperone activity of the Hsp104/Hsp70 bi-chaperone system in wild type cells is sufficient to reduce S100A8 and S100A9 amyloid toxicity and preserve cellular proteostasis. Expression of human *S100A8* and *S100A9* in yeast thus provides a novel model system for the study of the interaction of amyloid deposits with the proteostasis machinery.

## Introduction

S100 proteins are a family of 10- to 14-kDa EF-hand calcium-binding proteins that regulate diverse cellular processes affecting cell survival, proliferation, differentiation, and motility [1]. Marked changes have been observed in the expression levels of many S100 proteins in different types of cancer, neurodegenerative disorders, and inflammatory and autoimmune diseases. Among these proteins, S100A8 and S100A9, in particular, are involved in inflammation and cancer [2]; these two proteins activate the MAP kinase and NF-κB signaling pathways, trigger translocation of the RAGE receptor in human prostate cancer cells [3], and activate Toll-like receptor 4 [4], [5]. The secretion of S100A8 and S100A9 in response to cell damage or immune response activation constitutes a danger signal that activates immune and endothelial cells. Consequently, S100A8 and S100A9 are defined as damage-associated molecular pattern proteins in innate immunity [6], [7].

Structural studies indicate that *in vitro* S100A8 and S100A9 form homo- and hetero-dimers and heterotetramers [8]. This oligomerization is calcium dependent, and the binding of Ca^2+^ to the EF-hand domain triggers conformational changes that modulate the functional properties of S100A8 and S100A9 and their interactions with various molecular targets. Binding of Zn^2+^ to S100A8 and S100A9 leads to fine-tuning of their folding and may affect their function. The assembly of S100A8 and S100A9 into multiple hetero-dimeric and hetero-tetrameric complexes is considered to be a generic mechanism of protein functional diversification through variation of their conformational states and may determine their association with different ligands.

Nine different S100A proteins (A1–A6, A8, A9 and A12) appear in corpora amylacea inclusions in the brain during normal aging [9]. Recently, S100A8 and S100A9 were identified in amyloid aggregates in corpora amylacea of prostate cancer patients. That study was the first report of amyloid fibril formation by members of the S100 protein family. S100A8/9 inclusions were found in the corpora amylacea together with bacterial DNA and proteins that were surrounded by inflamed tissues infiltrated by neutrophils [10], [11]. The findings that under inflammatory conditions the S100A8/9 complex accounted for up to 40% of the total cytosolic proteins in neutrophils and that secreted S100A8/9 was found at high concentrations in inflamed tissues [12] has led to the hypothesis that S100A8/9 amyloids may be formed in response to chronic inflammation [10] and consequently may enhance the risk of cancer.

Accumulation of amyloid aggregates in cells is a common molecular event in a large number of human diseases [13]. Such diseases include Alzheimer’s disease, which is associated with the polymerization of amyloid β-peptide, and polyglutamine diseases, such as Huntington’s disease, which is characterized by the presence of extensions of the polyQ stretches in certain proteins [14]. Accumulation of misfolded proteins in the cell disrupts cellular homeostasis and can lead to toxicity and cell death.

Cells have developed an elaborate machinery to preserve protein homeostasis that involves several strategies aimed at either refolding, degrading, or sequestering misfolded proteins [15]. The cellular proteostasis machinery consists of molecular chaperones and of the cellular protein degradation machineries, such as the ubiquitin-proteasome system and autophagy pathways. Under optimal conditions, these mechanisms balance the cellular load of metastable and misfolded proteins [16], [17]. However, the expression of aggregation-prone proteins can interfere with the degradation of proteasome substrates, alter the subcellular distribution of essential proteins such as chaperones, and have deleterious effects on folding of other proteins [18], [19], [20], [21], [22]. It has been proposed that protein-misfolding diseases are initiated by the global disruption of cellular proteostasis due to the depletion and redistribution of essential components of the proteostasis network, resulting in a reduced protein folding capacity [23], [24].

The development of non-mammalian models to study protein aggregation diseases has been invaluable for the discovery of pathways and modifiers and for the elucidation of the underlying mechanism of toxicity [22]. Yeast has emerged as a simple eukaryote model for the characterization of amyloidogenic proteins and their interactions with cellular defense mechanisms [25], [26], [27], [28]. To examine the interactions of the aggregation-prone human S100A8 and S100A9 proteins with the proteostasis network, we established a novel model system by expressing them in the yeast, *S. cerevisiae.* Our current study showed that expression of the amyloidogenic human proteins, S100A8 and S100A9, in yeast does not affect the viability of wild type cells. Yeast, therefore, provides an excellent cellular model to specifically study the effect of aggregation of S100A8 and S100A9 proteins on the vital components of the cell proteostasis machinery. Indeed, we found that expression of S100A8 and S100A9 exposed unrelated metastable proteins in the background, which suggests that the expression of aggregating proteins significantly burdens the cell proteostasis mechanisms and can be a critical factor in their survival under stress conditions. We attribute increased toxicity of metastable proteins to depletion of molecular chaperones required for stabilization of the endogenous mutant protein.

## Results

### A Yeast Model for Investigating Human S100A8 and S100A9 Amyloidogenic Proteins

**Figure 1:**
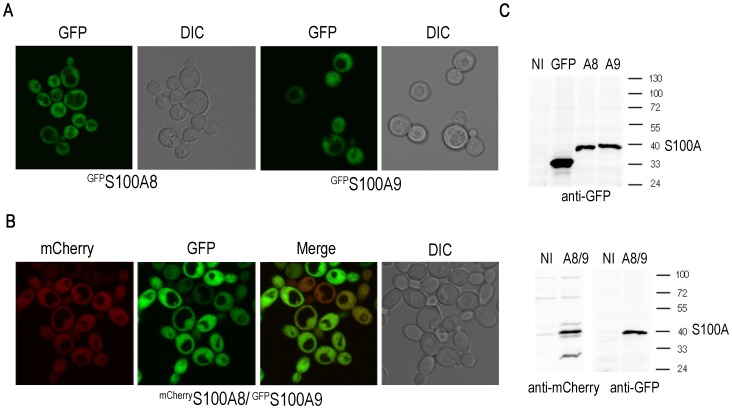
Overnight induction of p*GFP-S100A8* or p*GFP*-*S100A9* in yeast. (A) Single S100A8 or S100A9 yeast transformants or (B) cotransformants with plasmids ^mCherry^S100A8/^GFP^S100A9 were grown overnight in SG, and images were obtained with a fluorescence microscope. (C) TCA precipitates of extracts from cells growing on glucose or galactose medium were separated by 10% SDS-PAGE and analyzed by Western blot.

^GFP^S100A8 and ^GFP^S100A9 aggregates revealed an annular or punctate localization after two days of induction in wild type yeast (Figure 2A). Prolonged induction resulted in accumulation of S100A8 foci, specifically in the vacuole, as visualized by the FM4-64 lipophilic fluorescent dye [29] (34.4±4.5% compared with 16.7±0.7% for the GFP control, p<0.05). In contrast, ^GFP^S100A9 aggregates were observed throughout the cell after four days of induction (Figures 2B and 2C). Cotransformants, ^mCherry^S100A8/^GFP^S100A9, showed early formation of bright foci that were localized inside the vacuole of the cells (24.4±7.7% vacuolar compared with 9.7±4.8% cytoplasmic) after two days of induction, suggesting that S100A8 affected localization of the foci in the cotransformants (Figure 3 and data not shown). To support our observation that ^GFP^S100A8 foci accumulate in the vacuole, we produced ^GFP^S100A proteins in a *pep4Δ* deletion strain that lacks the vacuolar protease A [30] and examined the formation of foci. This treatment resulted in a sharp increase in ^GFP^S100A8 foci in the vacuole (80.9±4.1%) compared with the GFP control or with ^GFP^S100A9 (39.9±6.9% and 43.6±0.8%, respectively, p<0.005) (Figure 4). Thus, ^GFP^S100A8 and ^GFP^S100A9 transformed separately or together resulted in the formation of foci over time; both foci containing ^GFP^S100A8 alone and ^GFP^S100A8 co-transformed with ^GFP^S100A9 showed specific accumulation in the vacuole.

We established a *S. cerevisiae* model system to study human S100A8 and S100A9 protein aggregation and potential toxicity by expressing *S100A8* and *S100A9* from the inducible *GAL* promoter. S100A8 and S100A9 proteins were produced either as fluorescently tagged (p*mCherry-S100A8* or p*GFP-S100A8,* and p*GFP-S100A9,* respectively) or as non-tagged proteins. The plasmids were transformed separately (GFP-tagged proteins) or together (p*mCherry-S100A8* and p*GFP-S100A9)* into W303 wild type yeast and plated on either glucose (non-inducing conditions) or galactose (inducing conditions) plates. After overnight induction, cells transformed with p*GFP-S100A8*, p*GFP*-*S100A9* or with both p*mCherry-S100A8* and p*GFP-S100A9* showed diffuse fluorescence throughout the cytoplasm (Figure 1A and 1B). Western blot analysis of yeast that produced either one or both of the S100A proteins showed a protein band of the expected size in the induced cells but not in an empty vector control (Figure 1C).

**Figure 2:**
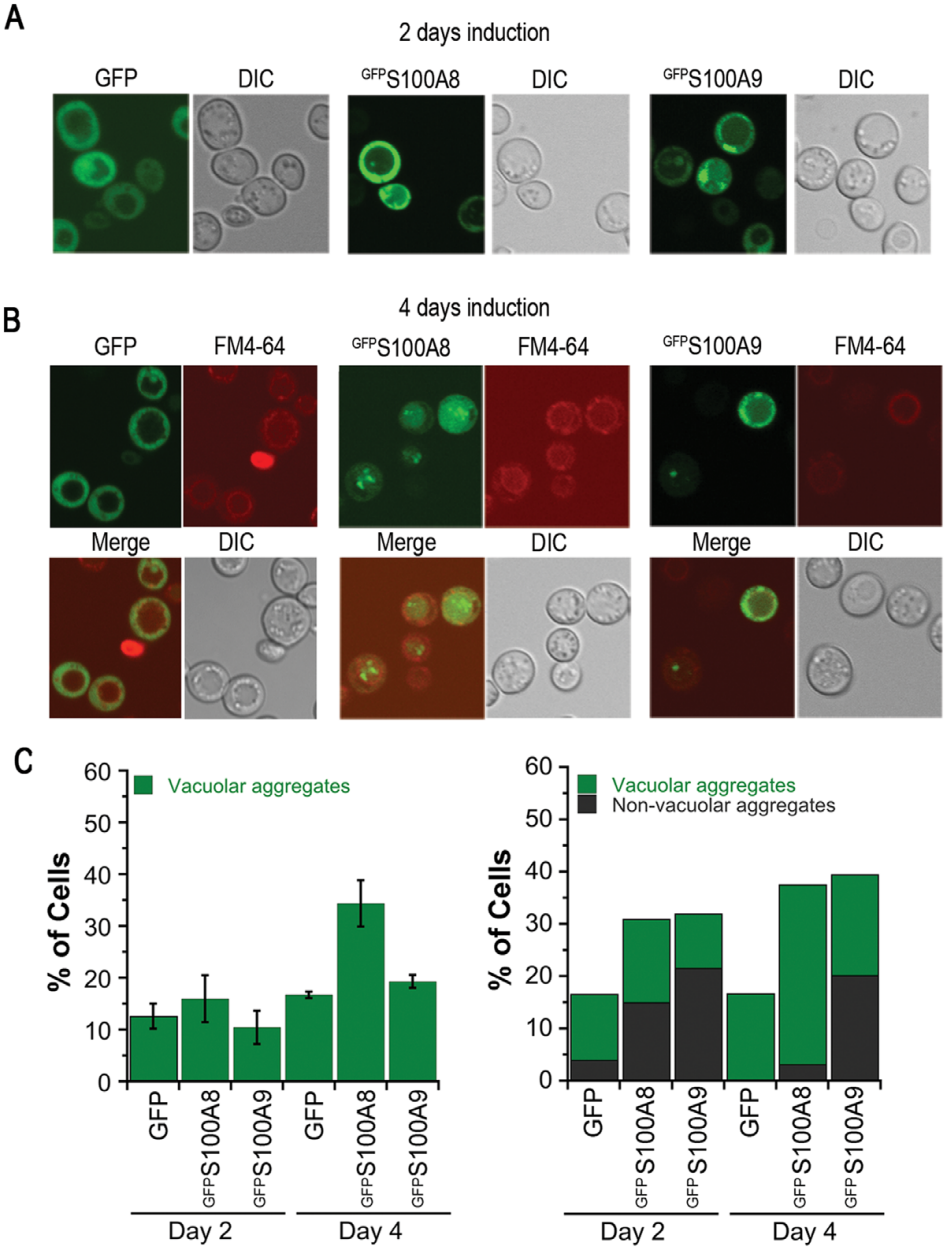
S100A8 and S100A9 form visible foci in yeast cells. Fluorescent microscope images of ^GFP^S100A8 and ^GFP^S100A9 (green) after (A) 2 days or (B) 4 days of induction. Lipophilic dye FM4-64 was used to visualize vacuoles (red). (C) Quantification of the percent of cells with GFP, ^GFP^S100A8 or ^GFP^S100A9 foci in the vacuole or in the cytoplasm, following 2 and 4 days of induction.

**Figure 3:**
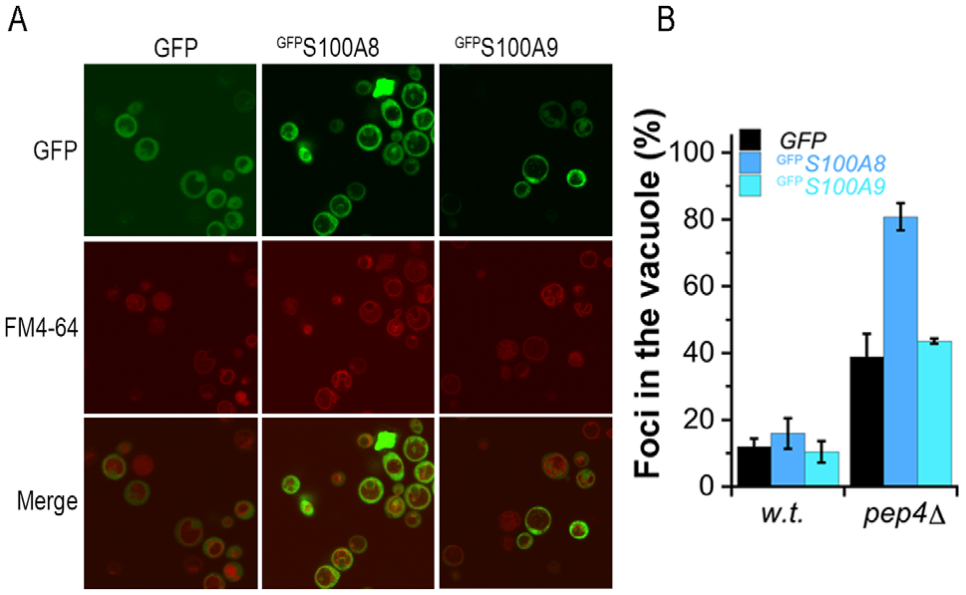
S100A8/9 form aggregates in yeast cells. GFP (green) and mCherry (red) fluorescent microscope images of ^mCherry^S100A8/^GFP^S100A9 cotransformed cells after 2 or 4 days of induction.

**Figure 4:**
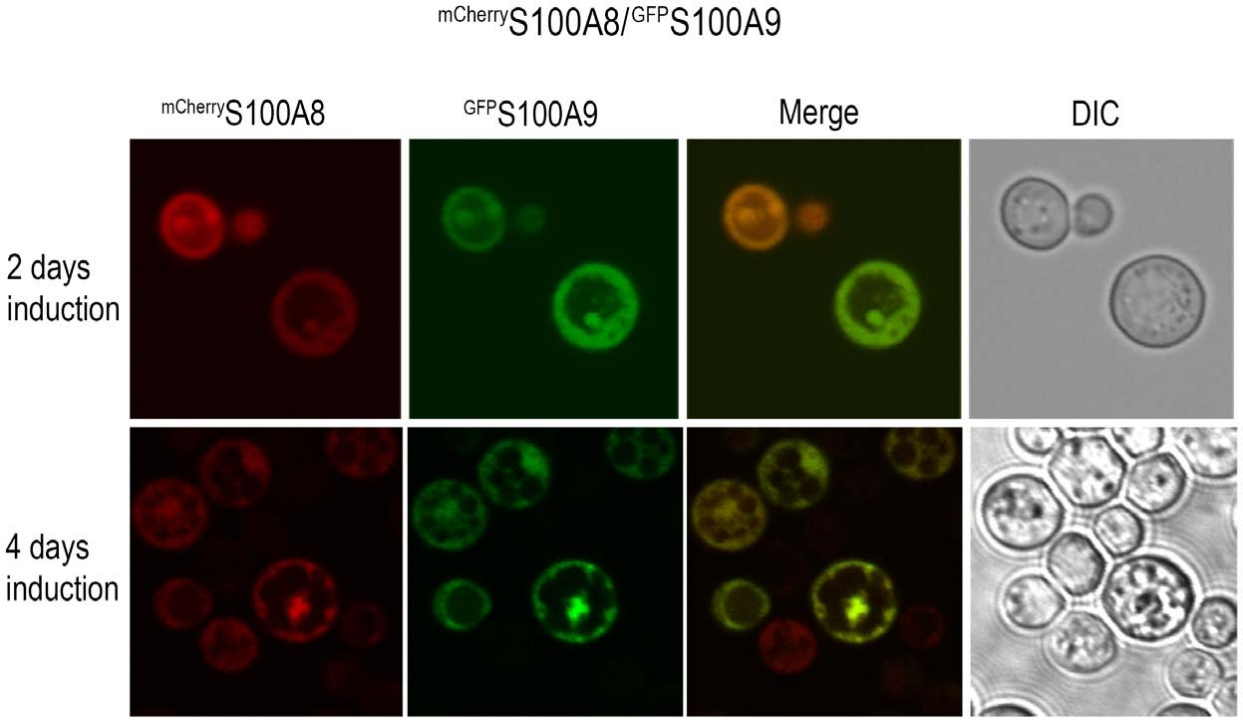
S100A8 accumulates foci in the vacuole in *pep4Δ* mutant cells. (A) Confocal images of GFP, ^GFP^ *S100A8* or ^GFP^ *S100A9* (green) after 2 days of induction in *pep4Δ* mutants cells. Lipophilic dye FM4-64 was used to visualize vacuoles (red). (B) Quantification of the percent of cells with GFP, ^GFP^S100A8 or ^GFP^S100A9 foci in the vacuole of *pep4*Δ cells, following 2 days of induction.

**Figure 5:**
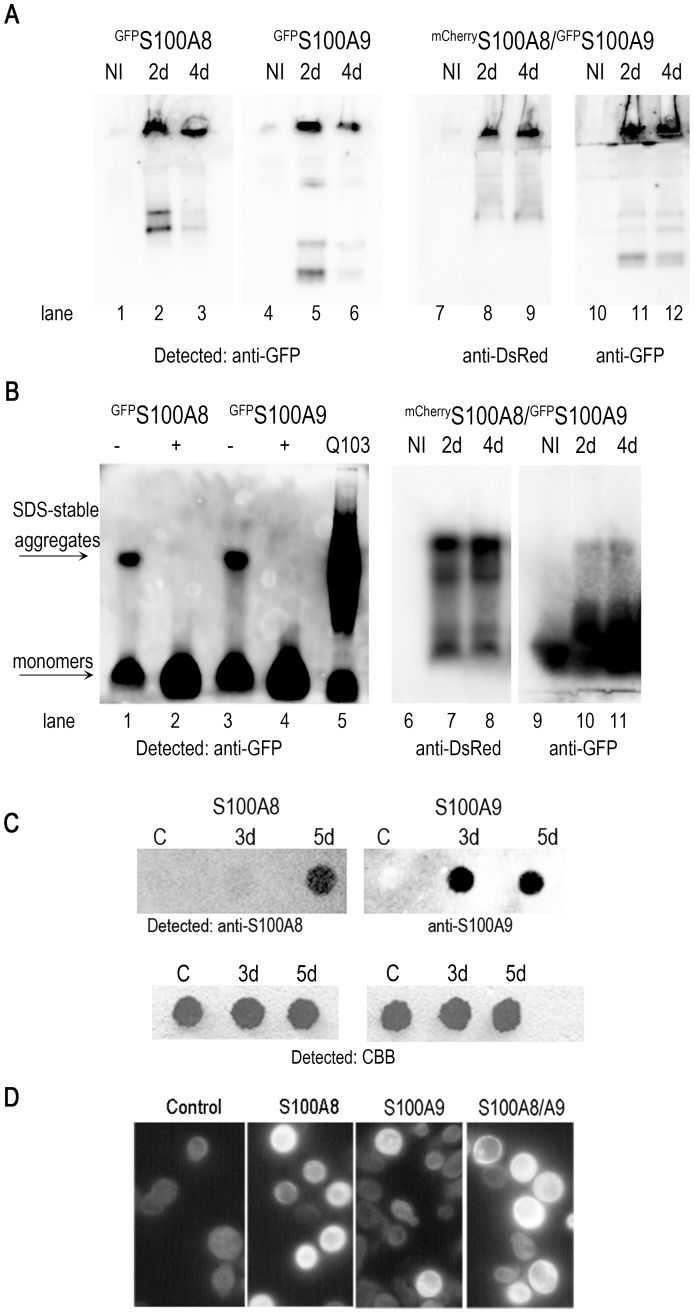
Aggregate formation by yeast cells transformed with p*GFP-S100A8*, p*GFP-S100A9* or p*mCherry-S100A8/*p*GFP-S100A9* after prolonged induction. (A) NI, noninduced control (Lanes 1, 4, 7, 10). Extracts of cells induced for 2 or 4 days to produce^ GFP^S100A8 (Lanes 2 and 3), ^GFP^S100A9 (Lanes 5 and 6) and ^mCherry^S100A8/^GFP^S100A9 (Lanes 8, 9 and 11,12) were separated on a native gel and analyzed by Western blot. (B) Semi-denaturing agarose detergent gel. After 2 days of induction ^GFP^S100A8 (Lane 1), ^GFP^S100A9 (Lane 3) or cotransformants ^mCherry^S100A8/^GFP^S100A9 (Lanes 7, 8 and 10, 11) formed SDS-stable aggregates in yeast cells. Boiling (+) the samples led to full soloubilization of aggregates to the monomeric form (Lanes 2 and 4). Total cell extracts (180 µg) were resolved using SDD-AGE. Blots were probed with anti-GFP or mCherry antibodies. Total cell extract of Q103^GFP^ cells (90**µg) was prepared after 24 h of induction (lane 5). (C) Filter retardation assay of cells grown for 3 and 5 days under inducing conditions. Loading control was visualized by CBB staining. Empty vector-transfected cells were used as control. (D) Spheroplasts of control and induced cells stained with ThT after 3 days of incubation on galactose plates.

The formation of very bright foci or ring-like structures is known to be strongly associated with ordered amyloid-like protein aggregation [25]. Given that S100A8 and S100A9 proteins form oligomeric and fibrillar structures [6], [8], [10], we examined their aggregation by native gel analysis. After two and four days’ induction S100A8 and S100A9 proteins formed insoluble high molecular weight (MW) structures that were retained in the well of the gel, indicative of aggregate formation (Figure 5A). Similar behavior was observed for the S100A8/9 co-transformation. High MW species were also detected upon semi-denaturing detergent-agarose gel electrophoresis (SDD-AGE) [31], [32]. After two days of incubation, ^GFP^S100A8 and ^GFP^S100A9 and ^mCherry^S100A8/^GFP^S100A9 proteins formed SDS-resistant aggregates that could be dissolved only by boiling (Figure 5B). This behavior was not related to the fluorescent tag, since non-tagged S100A8 and S100A9 proteins also formed insoluble aggregates on SDD-AGE gels (Figure S1) or in the filter-trap assay (Figure 5C). To further characterize the S100A aggregates, we used thioflavin T (ThT), a benzothiazole dye that exhibits enhanced fluorescence upon binding to β-sheets of protein amyloids both *in vivo* and in *vitro* [33]. After ThT staining of spheroplasts prepared from cells that expressed single non-tagged S100A8, S100A9 or both proteins, the S100A8 and S100A9 transformants exhibited bright fluorescence in addition to stained foci, similar to those observed with the fluorescently tagged proteins (Figure 5D). Thus, S100A8 and S100A9 protein expression in the yeast *S. cerevisiae* results in the formation of amyloid-like aggregates.

### S100A8 and A9 Aggregates are not Toxic in Yeast

**Figure 6:**
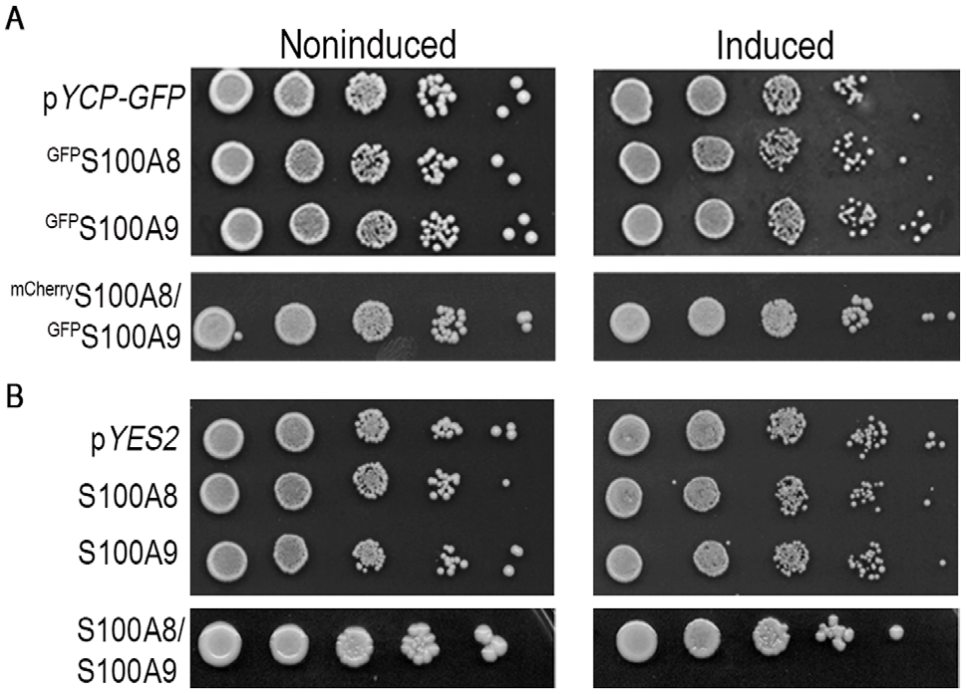
Viability of yeast cells expressing S100A8 and S100A9 proteins. (A) Ten-fold dilutions of yeast cells transformed with p*GFP-S100A8*, p*GFP-S100A9* or both plasmids were plated on glucose (non-inducing) or galactose (inducing) plates and photographed after 72 h. (B) Non-tagged proteins as in A.

Protein aggregation is often associated with growth arrest and cell death [25]. We therefore tested whether S100A8 and S100A9 are toxic to yeast. Serial ten-fold dilutions of cells expressing p*GFP-S100A8,* p*GFP-S100A9* or cotransformants with p*mCherry-S100A8* and p*GFP-S100A9* were plated on SD plates with either glucose or galactose. No marked effects on the viability of the cells expressing these two S100A proteins compared with those expressing an empty vector were observed (Figure 6A). Similar findings were obtained for non-tagged S100A8 and S100A9 (Figure 6B). Therefore, expression of S100A8 and S100A9 proteins in yeast led to the production of amyloid protein, with no effects on cell growth. This finding suggests that yeast cells can cope with the expression of the aggregation-prone S100A8 and S100A9 proteins and modulate their toxicity.

### S100A8 and S100A9 Aggregates are Toxic in the Presence of an Unrelated Metastable Protein

**Figure 7:**
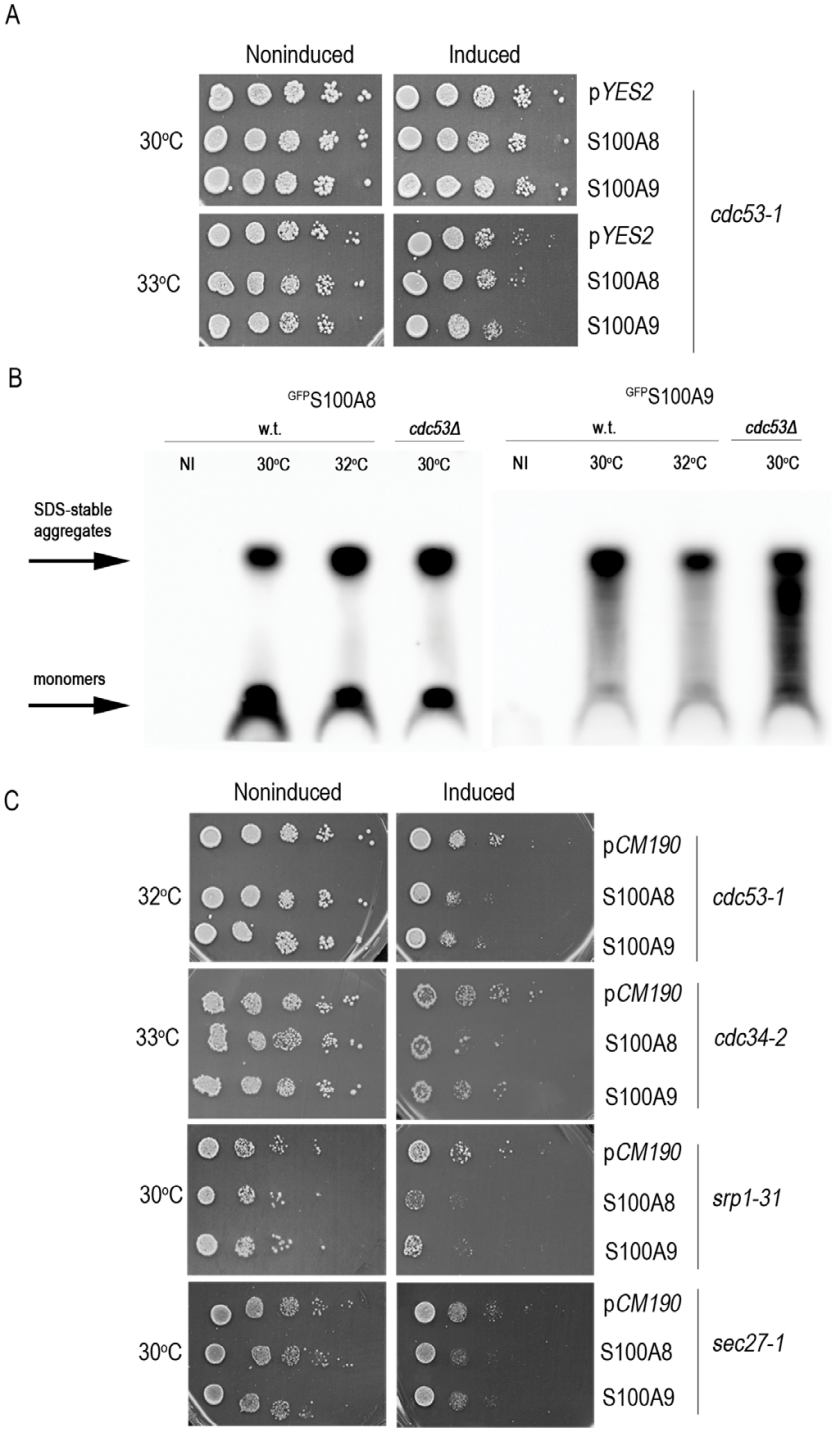
Viability of yeast cells producing S100A8 and S100A9 on the background of a *ts* mutation in an essential gene. (A) wild type and *cdc53-1* ts mutant cells expressing p*YES2-S100A8* or p*YES2-S100A9* were spotted on glucose or galactose plates and photographed after 72 h. (B**)** Semi-denaturing agarose gel. After 2 days of induction ^GFP^S100A9 forms aggregates in wild type and ts strain *cdc53-1* at 30°C and 32°C. Total cell extracts (180 µg) were resolved using SDD-AGE. (C**)** Ten-fold dilutions of *cdc53-1*, *cdc34-2*, *srp1-31,* and *sec27-1* yeast cells transformed with p*TET-S100A8* or p*TET-S100A9* were spotted on SD plates with (inducing) or without (non-inducing) 5 µg/ml doxycycline and photographed after 72 h.

Protein aggregation was shown to impact the cellular proteostasis machinery by inducing misfolding of temperature-sensitive (ts) metastable proteins under permissive conditions and exposing their specific phenotypes in a particular genetic background. This increase in protein misfolding load, in turn, enhanced protein aggregation [23], [24]. The extent and specificity of the genetic interaction varied with the characteristics of the aggregates formed [24]. Therefore, to assess the effects of S100A8 and S100A9 aggregation on cellular proteostasis, we examined their effects on the viability of cells with a ts mutation in an essential gene under permissive conditions. A ts *cdc53-1* mutant with a R488C substitution in the Cullin scaffold protein of the SCF (Skp1-Cullin-F-box protein) ubiquitin ligase complex required for cell cycle progression at the G_1_-S phase [34] was transformed with p*YES2-S100A8* or p*YES2-S100A9* (Figure 7A). Induction of *S100A8* or *S100A9* in the *cdc53-1* ts mutant resulted in growth inhibition at the permissive temperature of 33°C compared with cells transformed with an empty vector. These data indicate that S100A8 and S100A9 can uncover *cdc53-1* defects in viability at the permissive temperature. To examine whether *cdc53-1* can, in turn, affect S100A aggregation, we monitored the formation of high MW species using SDD-AGE. We found that at 30°C, a temperature at which no effect on cell viability was observed, expression of *S100A8* or *S100A9* in the *cdc53-1* mutant resulted in the accumulation of more high MW species than in the wild type strain (Figures 7B and S3).

*cdc53-1* mutant cells transformed with an empty vector alone showed a mild decrease in viability on galactose plates compared with glucose plates and this could have contributed to the reduction in viability observed when we induced the *S100A8* and *S100A9* genes with galactose. We therefore examined the toxicity of the ts mutant using an alternative inducible system in which *S100A8* and *S100A9* were regulated by the TET on-off promoter. There was no change in cell viability when wild type cells expressing p*TET-S100A8* or p*TET-S100A9* were grown on regular (inducing) or doxycycline-supplemented (non-inducing) plates (Figure S2A), although the proteins were expressed and formed aggregates, as visualized by ThT staining (Figure S2B). In contrast, expression of p*TET-S100A8* or p*TET-S100A9* in the *cdc53-1* mutant under permissive conditions (32°C) resulted in decreased viability (Figure 7C).

To extend our observations, we expressed *S100A8* and *S100A9* in three additional ts mutant strains: *cdc34-2*, a G58R mutant of the SCF-related ubiquitin-conjugating enzyme (E2) [35]; *srp1-31*, a S116F mutant of the importin-α ortholog that functions as a nuclear import receptor for proteins with a classic nuclear localization signal [36] *;* and *sec27-1*, a G688D mutant in an essential coat protein, involved in endoplasmic reticulum (ER)-to-Golgi and Golgi-to-ER transport [37]. Production of S100A8 or S100A9 in the *cdc34-2* mutant resulted in growth inhibition at 33°C, compared with cells transformed with an empty vector. Likewise, expression of *S100A8* or *S100A9* in the *srp1-31* or *sec27-1* ts strains resulted in S100A8- and S100A9-dependent toxicity at 30°C (Figure 7C). These results obtained with two different induction systems and with four different ts mutants indicate that S100A8 and S100A9 aggregation can differentially affect the function of an unrelated metastable protein in the cell and thus suggest that the capacity of the protein folding homeostasis machinery has been substantially reduced.

### Hsp104p Modulates S100A8- and S100A9-associated Toxicity

**Figure 8:**
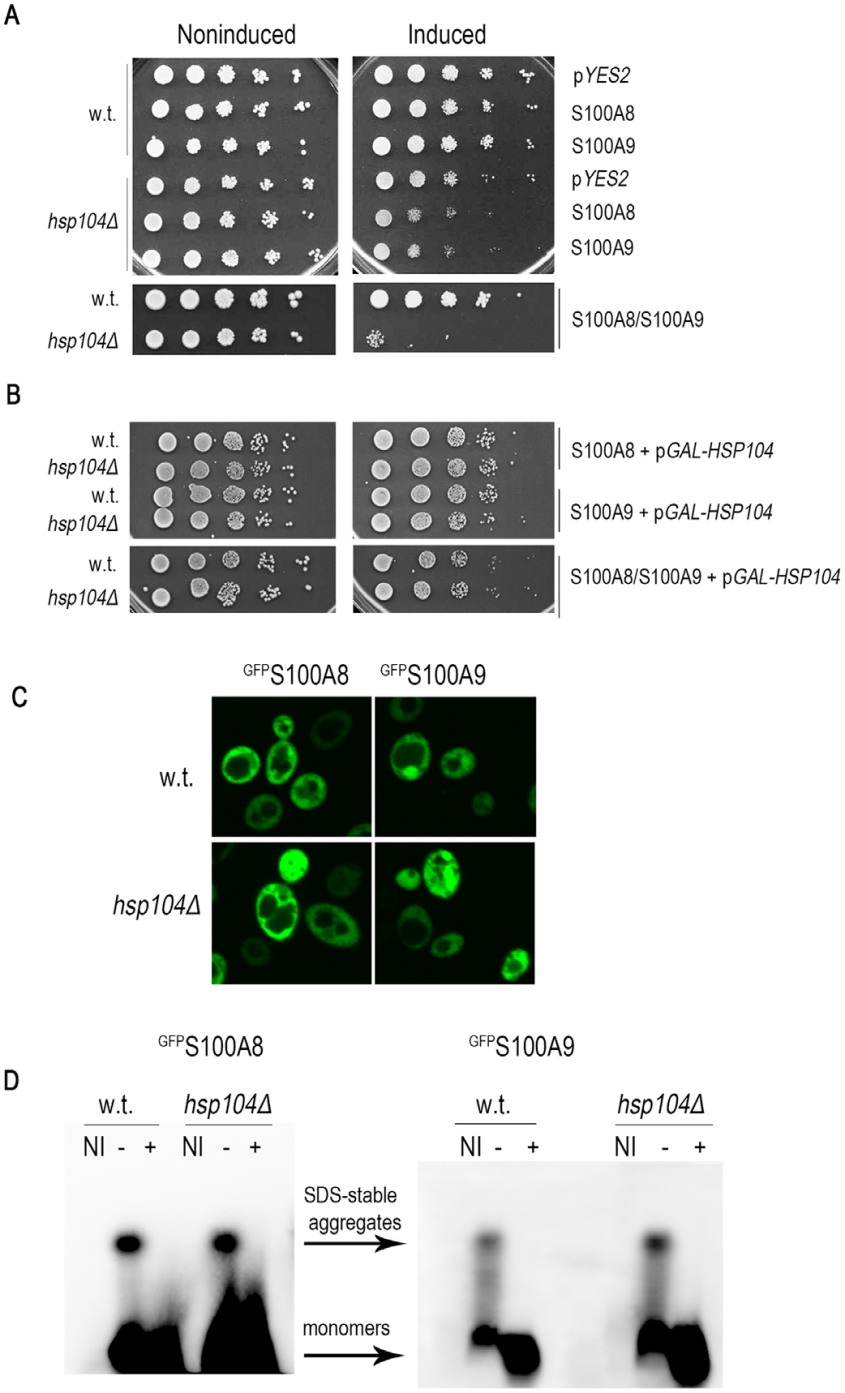
*HSP104* modulates S100A8- and S100A9-associated toxicity. (A) Viability of wild type or *hsp104Δ* yeast in the presence of S100A8, S100A9 and S100A8/9 proteins. p*YES2-S100A8*, p*YES2-S100A9* or both plasmids were expressed in wild type or *hsp104Δ* mutant cells. Viability was monitored using the spot test assay on inducing (galactose) or noninducing (glucose) plates. (B) Ten-fold dilutions of wild type cells or *hsp104Δ* mutants transformed with p*GALSc104(WT)* and with p*YES2-S100A8,* p*YES2-S100A9* or both S100 plasmids were plated on glucose (non-inducing) or galactose (inducing) plates. (C) Confocal images of ^GFP^S100A8 and ^GFP^S100A9 after 2 days of induction in wild type or *Δhsp104* mutant cells. (D) Cell extracts were prepared from wild type or *hsp104Δ* mutant cells expressing p*GFP-S100A8* or p*GFP-S100A9* after 2 days of induction. Extracts were incubated in 2% SDS sample buffer with (+) or without (−) boiling, loaded on agarose gels, and analyzed by Western blot using anti-GFP antibodies to detect the S100A8 and S100A9 proteins.

To directly examine the impact of S100A8 and S100A9 proteins on the cellular protein quality control capacity, we examined their genetic interactions with different components of the protein folding machinery. Whereas most chaperones bind to non-native or misfolded protein conformations that are generated when native proteins are denatured, for example, by stress, some chaperones, such as the heat-shock protein Hsp104, specifically interact with protein aggregates and can either promote or prevent amyloid formation and propagation of prions [38], [39], [40]. Yeast Hsp104p was found to act directly on protein aggregates and to lead to their resolubilization [41]. Therefore to examine whether Hsp104p is required for modulating S100A8 and S100A9 protein aggregation and toxicity, wild type and *hsp104Δ* mutants were transformed with plasmids p*YES2-S100A8*, p*YES2-S100A9* with or without p*GALSc104(WT)*, and the viability of the transformants was examined after galactose induction (Figures 8A and 8B). Induction of non-tagged S100A8 and S100A9 either individually or together led to a decrease of viability of *hsp104Δ* mutants compared with that of wild type cells (Figure 8A). Elevating the levels of Hsp104p in the *hsp104Δ* mutant, by overexpressing *Hsp104p* from the *GAL* promoter, restored viability, thereby supporting a role for Hsp104p in modulating S100A8 and S100A9 toxicity (Figure 8B). Both wild type cells and *hsp104Δ* mutants that produced tagged ^GFP^S100A8 and ^GFP^S100A9 proteins showed foci after two days of induction, indicating that protein aggregation was not affected (Figure 8C). To further examine how Hsp104p influences the aggregation status of ^GFP^S100A8 and ^GFP^S100A9 proteins, we monitored the accumulation of high MW species with SDD-AGE. After two days of induction, there were no significant changes in ^GFP^S100A8 and ^GFP^S100A9 high MW species in *hsp104Δ* mutants compared with wild type cells (Figures 8D and S4). Thus, whereas deletion of *HSP104* resulted in increased toxicity, it had little effect on aggregation. This finding suggests that Hsp104p is involved in modulating S100A8 and S100A9 toxicity and is required for cell viability.

### Hsp70p Chaperones are Required for Preservation of Cell Viability in the Presence of S100A8, S100A9, or S100A8/9 Aggregates

**Figure 9:**
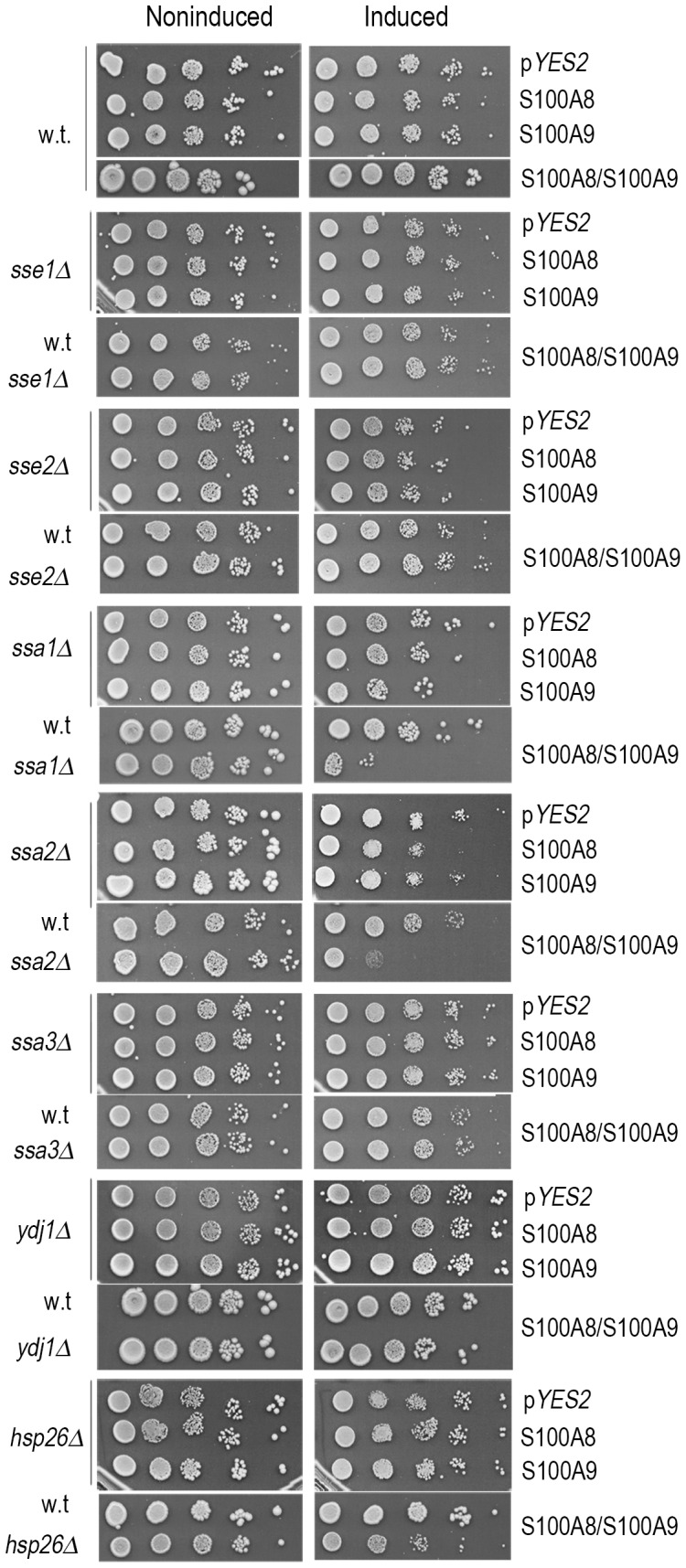
Viability of single chaperone deletion strains producing S100A8 and S100A9 proteins. Cells expressing empty vector (p*YES2),* p*YES2-S100A8*, p*YES2-S100A9*, or cotransformants with p*YES2-S100A8/*p*YES2-S100A9* in *sse1, sse2, ssa1, ssa2, ssa3, hsp26, and ydj1* mutants and the isogenic wild type parent were spotted on galactose and glucose plates and photographed after 72 h.

In addition to Hsp104p, several molecular chaperones have been reported to be involved in the disaggregation machinery in yeast; these include Hsp70p and Hsp40p *inter alia* [42], [43], [44]. We therefore examined which other chaperones are required for modulating S100A8 and S100A9 toxicity. Cells deleted for two of the *HSP70* chaperones (*ssa1Δ* and *ssa2Δ*) were transformed with p*YES2-S100A8,* p*YES2-S100A9* or both plasmids together, and cell viability after production of S100A8 and S100A9 proteins was examined. We found that deletion of either *SSA1* or *SSA2* led to a mild decrease in viability, which was aggravated in the cotransformants (Figure 9). This observation suggests that in addition to Hsp104p, yeast Hsp70p is also involved in modulation of S100A8 and S100A9 protein aggregation and toxicity. In contrast, the viability of the mutant deleted for *SSE1* or for *SSE2,* that encode nucleotide exchange factors for Hsp70p proteins, was not affected by production of S100A8 or S100A9. Similarly, deletion of *YDJ1* that encodes Hsp40p or of *HSP26* did not affect the viability of cells that produced S100A8 or S100A9 either separately or together in cotransformants (Figure 9). These results indicate that the Hsp104p/Hsp70p bi-chaperone system is involved in modulating S100A8 and S100A9 toxicity.

## Discussion

The budding yeast *S. cerevisiae* has been used as a model organism for many different neurodegenerative diseases, including Parkinson’s and Huntington’s diseases [21], [45], [27]. Here, *S. cerevisiae* was used as a model system for studying human S100A8 and S100A9 aggregation. ^GFP^S100A8 aggregates, both those formed initially in the cytoplasm and those formed after prolonged induction, showed pronounced vacuolar localization. In contrast, ^GFP^S100A9 aggregates were found predominantly in the cytoplasm but could be localized to the vacuole when co-expressed with S100A8, which suggests that similar proteins form distinct aggregates that are handled differently by the cellular quality control machinery. For example, a triple proline mutant of α-synuclein displayed a vacuolar phenotype characterized by an increased number of foci in the vacuole, whereas other α-synuclein mutants showed a different cellular distribution [30]. We did not detect differences in cell viability associated with localization of the S100A8 and S100A9 aggregates, despite our hypothesis that different folding and clearance mechanisms could be involved in aggregate modulation in different compartments of the cell.

The relationship between amyloid formation and amyloid toxicity in protein conformational diseases is not well understood, and it is not clear which step of the amyloid formation cascade is toxic. This step may vary for different amyloid diseases [46]. Sequestration of aggregates into inclusion bodies may be a cellular defense mechanism [47], [48], [49]. For example, in amyotrophic lateral sclerosis TDP-43 protein, a component of neuronal aggregates, was not associated with toxicity when sequestered into inclusion bodies, but was a strong and independent predictor of neuron cell death when located in the cytoplasm [50]. Likewise, the majority of neurons expressing mutant huntingtin died without forming inclusion bodies, thus linking the formation of aggregates with survival of neurons [51]. Here, prolonged induction and high levels of the S100A8 and S100A9 proteins alone or together and their subsequent aggregation did not affect the viability of wild type yeast. Our study, therefore, supports the observation that there is no direct correlation between protein aggregation and cell viability.

The formation of inclusion bodies in Huntington’s disease was shown to partially restore longevity by improving the ubiquitin proteasome system throughput and consequently lowering the overall cellular burden of misfolded proteins [52]. These results suggest that the pathogenic mechanism of amyloidosis could, in part, result from an imbalance in protein folding homeostasis [53]. Overwhelming proteostasis, for example, in the presence of an unrelated ts metastable protein can enhance the misfolding of polyQ-containing proteins [23] and probably also of other amyloid-prone proteins. Indeed expression of *S100A8* and *S100A9* in mutants with metastable essential proteins, *cdc53-1, cdc34-2*, *srp1-31* or *sec27-1* ts mutants of yeast, led to a reduction in cell viability. We suggest that high levels of these protein aggregates perturb the cellular protein homeostasis mechanisms and that the molecular chaperones required for maintaining these essential cell cycle proteins in a functional conformation are depleted due to their recruitment to the S100A8 or S100A9 aggregates. Thus, our finding that S100A8 and S100A9 aggregation can differentially affect the function of unrelated metastable proteins in the cell suggests that the capacity of the protein folding homeostasis machinery has been compromised. It is possible that the general load of damaged proteins on the cell – rather than the specific folding or clearance machinery involved – determines cellular toxicity.

Molecular chaperones control almost all aspects of cellular proteostasis [54], [55]. Most chaperones facilitate protein folding and prevent protein aggregation. The influence of the chaperone network on protein aggregation in general and of S100A8 and S100A9 in particular are far from clear. Here, we have demonstrated how the chaperone machinery can modulate amyloid formation and toxicity of S100A8 and S100A9 proteins. We examined several chaperones that play a role in other human amyloidosis conditions and are involved in modulation of prion propagation and toxicity [42], [56]. The effect of Hsp104p, one of the main players in the disaggregation machinery, on protein misfolding events has been studied in different yeast models of amyloid diseases. Deletion of *HSP104* was shown to eliminate polyQ aggregation and toxicity [57], and inhibition or elevated levels of Hsp104p cured several yeast prions [58], [59]. Furthermore, overexpression of *HSP104* solubilized SDS-resistant polyQ aggregates and greatly reduced toxicity. In contrast, overexpression of *HSP104* in an α-synuclein yeast model did not show any significant effect on aggregation and toxicity [60]. Thus, the ability of the Hsp104p chaperone to suppress toxicity of aggregates seems to depend on the nature of the aggregated protein. Our results indicate that in the S100A8 and S100A9 model, the Hsp104p protein modulates the toxicity of amyloid aggregates and plays a protective role in the yeast cells. Deletion of *HSP104* may lead to formation of toxic oligomeric forms that decrease cell viability. Expression of *HSP104* in *hsp104Δ* mutants in the presence of S100A8 and S100A9 proteins led to restoration of viability. This finding suggests that in wild type cells the levels of Hsp104p are sufficient to modulate S100A8 and S100A9 toxicity and maintain cell viability.

Hsp104p usually requires other chaperones to function; in particular, Hsp104p collaborates with Hsp70p and Hsp40p to disaggregate protein amyloids [61]. Other small heat shock proteins, such as Hsp26p, have been shown to contribute to the clearance of aggregates by Hsp104p [60], [62]. Several cytosolic forms of Hsp70p have been implicated in the disaggregation process in yeast. Different Hsp70 proteins were shown to affect aggregation of prion and other amyloidogenic proteins differently in yeast models. For example, mutation in *SSA2* destabilized the [URE3] prion, whereas deletion of *SSA1* did not affect prion propagation [63], [64]. In Huntington’s disease, polyQ toxicity was decreased in a double mutant of *ssa1*Δ, *ssa2*Δ [65]. In our S100A8–S100A9 yeast model, members of the Hsp70p family, Ssa1p and Ssa2p, were found to protect cells from toxicity caused by S100A8 and S100A9 amyloids. Deletion of *SSA1* and *SSA2* in the presence of S100A8 or S100A9 alone was mildly toxic to the yeast cells. In contrast, a strong toxic effect was observed when both S100A8/9 proteins were produced in the same mutant cell. Our data indicate that in the absence of other single molecular chaperones, such as Hsp26p, Ydj1p, Sse1p, and Sse2p, S100A8 and S100A9 aggregates had no effect on cell viability. However, we cannot exclude the possibility that these chaperones may have redundant roles in modulating S100A8 and S100A9 toxicity.

In summary, we have clearly demonstrated that S100A8 and S100A9 proteins form non-toxic amyloid aggregates in yeast cells when expressed either alone or together. However, cells carrying the burden of amyloid aggregates become hypersensitive to the presence of metastable proteins, such as mutants of Cdc53, Srp1, Cdc34, and Sec27, because the overall protein folding machinery becomes exhausted and compromised. The presence of S100A8 and S100A9 aggregates unmasks the weaker parts in cellular regulation and the interconnection between vital components of the protein network that maintains cell viability. We found that yeast cells that produce S100A8 and S100A9 aggregates require the Hsp104/Hsp70 bi-chaperone machinery as essential factors for preservation of cell viability. Thus, we have developed a very powerful and sensitive model to study the effect of S100A8 and S100A9 aggregation on the functioning of the cell homeostasis machinery and to examine their effect on key components of the protein folding and aggregate clearing systems. Considering that human S100A8 and S100A9 amyloids are broadly associated with inflammation and also with age-dependent deposits, our cellular model can provide a platform for further investigations to identify other members of the protein homeostasis machinery that determine the toxicity of these amyloids.

## Materials and Methods

### Yeast Strains

Wild type W303 (MAT**a**, ade2Δ1, ura3-52, trp1Δ2, leu2-3,112, his3-11) [66]; BY4741 (MAT**a**, his3Δ1, leu2Δ0, met15Δ0, ura3Δ0), (Euroscarf). cdc34-2 (MAT**a,** ura3-52, leu2-2, bas1-2, bas2-2, GAL2+, gcn4-1, ade8::GCN4, trp1-1, cdc34-2) is a ts strain with a G58R substitution mutation [35]. cdc53-1 (MAT**a,** ura3-1, can1-100, GAL^+^, leu2-3,112, trp1-1, his3-11,15, ade2-1, cdc53-1) was obtained from M. Tyers and has a R488C substitution mutation [34]. srp1-31 (MAT**α**, srp1-31, ura3, leu2, trp1, his3, ade2) was obtained from J. Hood and has a S116F substitution mutation [67]. sec27-1 (MATα, leu2-3,112, trp1, ura3-52, sec27-1) was obtained from J. Gerst and has a G688D substitution mutation [68]. Strains with deletion of HSP104, HSP40, HSP26, SSE1, SSE2, SSA1, SSA2, and SSA3 were obtained from the Euroscarf deletion library (MAT**a**, YYYΔ::Kan^r^ (KO/DAmP), his3Δ1, leu2Δ0, met15Δ0, ura3Δ0, CAN1, LYP1).

### Yeast Plasmids

*Plasmids for expression of S100A8 and S100A9 with fluorescent tags:* The *S100A8* and *S100A9* genes were amplified from *Escherichia coli* PET1120 plasmids that encoded each of these human genes [69] using primer pair A8SalIF/A8XhoIR for S100A8 and A9BamHIF/A9HinDIIIR for S100A9 (Table S1). Each PCR product was cloned into pGEM T-easy (Promega). p*mCherry-S100A8* was constructed by amplifying mCherry from plasmid PCS2-mCherry [70] with primer pair mCherryEcoRIF/mCherrySalIR and cloning the PCR product into pGEM T-easy. The mCherry fragment was extracted with EcoRI and SalI and ligated with the above S100A8 fragment cleaved with XhoI and EcoRI. The mCherry-S100A8 fragment was cloned as an EcoRI fragment into pYES2 (Invitrogen). p*GFP-S100A8* was constructed by amplifying *S100A8* from Pet1120 using primer pair A8BamHIF/A8HinDIIIR, followed by insertion into pGEM T-easy and subsequent transfer as a BamHI-HinDIII fragment into p*YCPGAL-GFP* [71]. Similarly, the *S100A9* PCR product was cloned into pGEM T-easy and then transferred as a BamHI-HinDIII fragment into p*YCPGAL-GFP*.

*Plasmids for expression of non-tagged S100 proteins* ***:*** *S100A8* and *S100A9* cloned into pGEM T-easy as above were excised with HinDIII and XhoI (*S100A8*) and with BamHI and EcoRI (*S100A9*) for cloning into p*YES2*. The plasmid marker gene, *URA3*, was converted to *LEU2 in vivo* by homologous recombination using p*YIp1392* in the case of *pYES2-S100A8* to enable cotransformation of yeast cells with both plasmids. p*CM190* was used for expression of S100A8 and A9 under the TET on-off promoter [72]. *S100A8* and *S100A9* were subcloned with a pair of restriction enzymes as a BamHI and HinDIII fragment from p*GFP-S100A8* and p*GFP-S100A9*, respectively.

*The plasmid used for overexpression of HSP104*: p*GALSc104(WT)* was purchased from Addgene (plasmid number 1146) [73], and p*103Q-GFP* was as described [65]. Primer sequences appear in Table S1. Plasmids are listed in Table S2.

### Yeast Transformation and Growth Conditions

Yeast cultures were grown overnight at 30°C (28°C for ts strains) in a rotary thermoshaker at 120 rpm in synthetic minimal medium (SD) supplemented with the appropriate amino acids. For constructs under the *GAL* promoter, SD medium and 2% galactose (SG) medium were used for non-inducing and inducing conditions, respectively. For aggregate detection, yeast strains were grown at 30°C for two to six days on selective SG agar plates. For constructs under the TET on-off promoter, SD medium with 5 µg/ml doxycycline and synthetic 2% glucose (SD) medium were used for non-inducing and inducing conditions, respectively. For aggregate detection, yeast strains were grown at 30°C for two to six days on selective SD agar plates. Yeast transformation was as in [74].

### Spot Test Viability Assay

Yeast cells were grown overnight in SD medium or in synthetic medium with 2% raffinose, diluted 1∶3, and then regrown to mid-log phase. Equal amounts of cells at OD_600_ = 0.5 were harvested by centrifugation at room temperature for 2 min at 1500g, washed three times in sterile water, and then resuspended in sterile water. Ten-fold serial dilutions were made in sterile water, and 5-µl drops were plated on SD or SG medium complemented with the appropriate amino acids_._ The plates were incubated for two to four days at 30°C. For ts strains, yeast cells were grown at 28°C under non-inducing conditions, washed, and transferred to appropriate medium for induction. Cells were then plated on SD medium or SD with 5 µg/ml doxycycline medium and incubated at 30–33°C, as indicated in the text.

### TCA Precipitation

Yeast cells were resuspended in 20% TCA and incubated on ice. Glass beads were added, and the cells were broken by vigorous vortex for 3×5 min at 4°C. Precipitates were collected by centrifugation at 14000 g for 10 min. Pellets were dissolved in 50 µL of dissociation buffer (4% sodium dodecyl sulfate, 0.1 M Tris-HCl [pH 6.8], 20% glycerol, 2% 2-mercaptoethanol, 0.02% bromophenol blue) and 25 µl of 1 M Tris-base. Yeast extracts were incubated for 5 min at 100°C and separated on SDS-PAGE. We used a nitrocellulose membrane (Biorad, 0.45 µm) for transfer and detection.

### Antibodies

Mouse anti-GFP (Santa Cruz Biotechnology) and rabbit anti-mCherry (Clontech) at 1∶1000 dilutions were used for Western blot analysis of tagged proteins. For detecting non-tagged proteins, we used monoclonal and polyclonal anti-calgranulin A (S100A8) and anti-calgranulin B (S100A9) (Santa Cruz Biotechnology) at 1∶100 dilution. Goat anti-mouse (1∶2500) and anti-rabbit (1∶5000) antibodies were purchased from Sigma. As a loading control we used anti-actin antibody (Sigma) at 1∶5000 or anti-GAPDH antibody (Abcam) at 1∶1000 dilutions.

### Native Gel

Yeast cells were grown for two to four days on either SD or SG selective plates. The cells were collected and washed with phosphate-buffered saline (PBS) and adjusted to OD_600_ = 0.8 in PBS. Then, 20 ml of cells were collected by centrifugation and transferred into 200 µl PBS with proteinase inhibitor (1∶1000). Glass beads were added, and the cells were broken as above. The cell debris and glass beads were removed by centrifugation at 100 g for 0.5 min. The extract was clarified by centrifugation at 650 g for 2 min and kept on ice before addition of sample buffer ×5 (50% glycerol, 0.5 Tris HCl pH 6.8, 0.02% bromophenol blue). For Western blot analysis, 40 µl of extract were dissolved in 10 µl of sample buffer, and 30 µl of each fraction was separated on a 12% native-PAGE gel with a 3.5% stacking gel, followed by Western blot analysis. The gels were run at 60 V for approximately 4–5 h at 4°C. The gel was transferred onto a polyvinylidene difluoride (PVDF) membrane at 300 A for 1.2 h in transfer buffer (Tris-glycine buffer with 10% methanol and 0.1% SDS) and analyzed by Western blotting with the antibodies described above.

### SDD-AGE

For semi-denaturing agarose gel electrophoresis, yeast cells were broken by glass bead disruption under moderately denaturing conditions (50 mM NaCl, 100 mM Tris pH 7.5, 10 mM β-mercaptoethanol, complete protease inhibitor cocktail without EDTA (Roche) –1 tablet for 25 ml). Yeast extracts were cleared by centrifugation (650 g, 2 min, 4°C). Protein concentrations of the samples were normalized at 2.0 mg/ml. Normalized extracts (150–200 µg) were added to 4 × SDD–AGE buffer to a final concentration (50 mMTris, pH 6.8, 2% (w/v) SDS, 0.025% bromophenol blue, 5% glycerol). Extracts were incubated at room temperature for 10 min and loaded onto a 1.5% agarose gel (prepared with 1× Tris-glycine buffer (20 mM Tris, 200 mM glycine +0.1% SDS). Samples were run in a regular horizontal gel chamber for DNA electrophoresis in running buffer (20 mM Tris, 200 mM glycine +0.1% SDS) [75] at 80 V until the bromophenol reached the bottom edge of the gel. Gels were transferred onto a PVDF membrane for analysis by Western blotting.

### Filter Trap Assay

Yeast cells were broken by glass bead disruption in extract buffer (50 mM NaCl, 100 mM Tris pH 7.5, 10 mM β-mercaptoethanol, complete protease inhibitor cocktail without EDTA (Roche) –1 tablet for 25 ml). Yeast extracts were cleared by centrifugation (650 g, 2 min, 4°C). The extracts were diluted 1∶3 in PBS buffer with 2% SDS and incubated at room temperature for 10 min or boiled for 5 min and then filtered through a 96-well dot blot apparatus (Bio-Rad Laboratories, Hercules, CA) containing nitrocellulose (0,2 µm, Bio-Rad) or cellulose acetate membranes (0,2 µm, Millipore) and analyzed by Western blotting. Total protein load was assessed by coomassie brilliant blue (CBB) staining of the nitrocellulose membrane.

### ThT Binding Assay

Each culture was grown on selective plates for three to four days and then diluted into liquid medium and grown to OD_600_ = 0.5. The cells were washed in 50 mM Phosphate buffer (pH 6.5) with 1 mM MgCl_2_. A 5-ml sample of the culture was transferred into freshly prepared 4% formaldehyde fixative in this buffer and incubated at room temperature for 1.5–2 h with brief vortexing every 30 min. The fixed cells were collected by centrifugation (2 min at 2000 g), and the supernatant was carefully removed. The cells were washed in 5 ml buffer, and the pellet was resuspended at OD_600_ = 10 in PMST buffer (0.1 M H_2_KPO_4_, pH 7.5; 1 mM MgCl_2_; 1.2 M sorbitol; 0.1% Tween 20; complete protease inhibitor cocktail without EDTA). For spheroplasting, 100 µl of the cell suspension were transferred to a 0.5-ml Eppendorf tube and incubated with 50–100 U Zymolyase (Zymo Research) on a rotating wheel at room temperature for 15 min. The spheroplasts were gently resuspended in 100 µl PMST, centrifuged and resuspended. The cells were washed with PBS, pH 7.4, and incubated in PBS with 0.001% ThT for 20 min, washed three times with PBS, and then observed immediately by fluorescence microscopy.

### Microscopy

*GFP-S100A8* and *GFP-S100A9* and *mCherry-S100A8/GFP-S100A9* were induced with 2% galactose. Imaging was performed with an Olympus FV1000 laser-scanning confocal microscope with a x60 objective lens. Fluorescence was excited with 543 nm for the red fluorescent markers and 488 nm for GFP.

### Staining with FM4-64

For staining with FM4-64 (Molecular Probes) after 2 and 4 days, cells expressing *^GFP^S100A8*, *^GFP^S100A9* or *^mCherry^S100A8/^GFP^S100A9* were collected from a SG-induced plate and incubated with 15 µM dye (5 mM FM4-64 in dimethyl sulfoxide was diluted with water to obtain a 150 mM stock solution) for 30 min in SG. Cells were washed three times with the same medium and immediately observed under a microscope.

## Supporting Information

Figure S1 **Analysis of S100A8 aggregates on SDD-AGE gels.** Cell extracts were prepared from wild type cells that produced S100A8 protein after 2, 4, and 6 days of incubation on galactose plates. The yeast extracts were incubated for 10 min in 2% SDS sample buffer at room temperature with (+) or without (−) boiling and then loaded on the SDD-AGE gel and analyzed by Western blot using polyclonal anti-S100A8 antibodies.
(TIF)

Figure S2 **Aggregation and toxicity of S100A proteins induced by a TET on-off promoter system.** (A) Ten-fold dilutions of wild type yeast cells transformed with p*CM190* (empty vector), p*TET-S100A8* or p*TET-S100A9* were plated on glucose (inducing) or glucose with 5 µg/ml doxycycline (non-inducing) plates. (B) Spheroplasts of control and induced cells stained with ThT after 4 days of incubation on glucose plates.
(TIF)

Figure S3 **S100A8 and S100A9 protein levels in wild type and** ***cdc53-1*** **cells.** Wild type or *cdc53-1* mutant cells expressing ^GFP^S100A8 or ^GFP^S100A9 were grown for 2 days on glucose or galactose plates at 30°C or 32°C. TCA precipitates of extracts were separated by 10% SDS-PAGE and analyzed by Western blot, using anti-actin or anti-GAPDH and anti-GFP antibodies.
(TIF)

Figure S4 **S100A8 and S100A9 protein levels in wild type and** ***hsp104Δ cells.*** Wild type or *hsp104Δ* mutant cells expressing *GFP-S100A8* or *GFP-S100A9* were grown for 2 days on glucose or galactose plates. TCA precipitates of extracts were separated by 10% SDS-PAGE and analyzed by Western blot, using anti-actin and anti-GFP antibodies.
(TIF)

Table S1 **Primer sequences.**
(DOC)

Table S2 **Plasmids for expression in yeast.**
(DOC)
