# Supplementary figures and images for: Aggregation of Human S100A8 and S100A9 Amyloidogenic Proteins Perturbs Proteostasis in a Yeast Model
Source: PLoS One. 2013 Mar 6;8(3):e58218. doi: 10.1371/journal.pone.0058218 (PMC3590125; doi:10.1371/journal.pone.0058218)

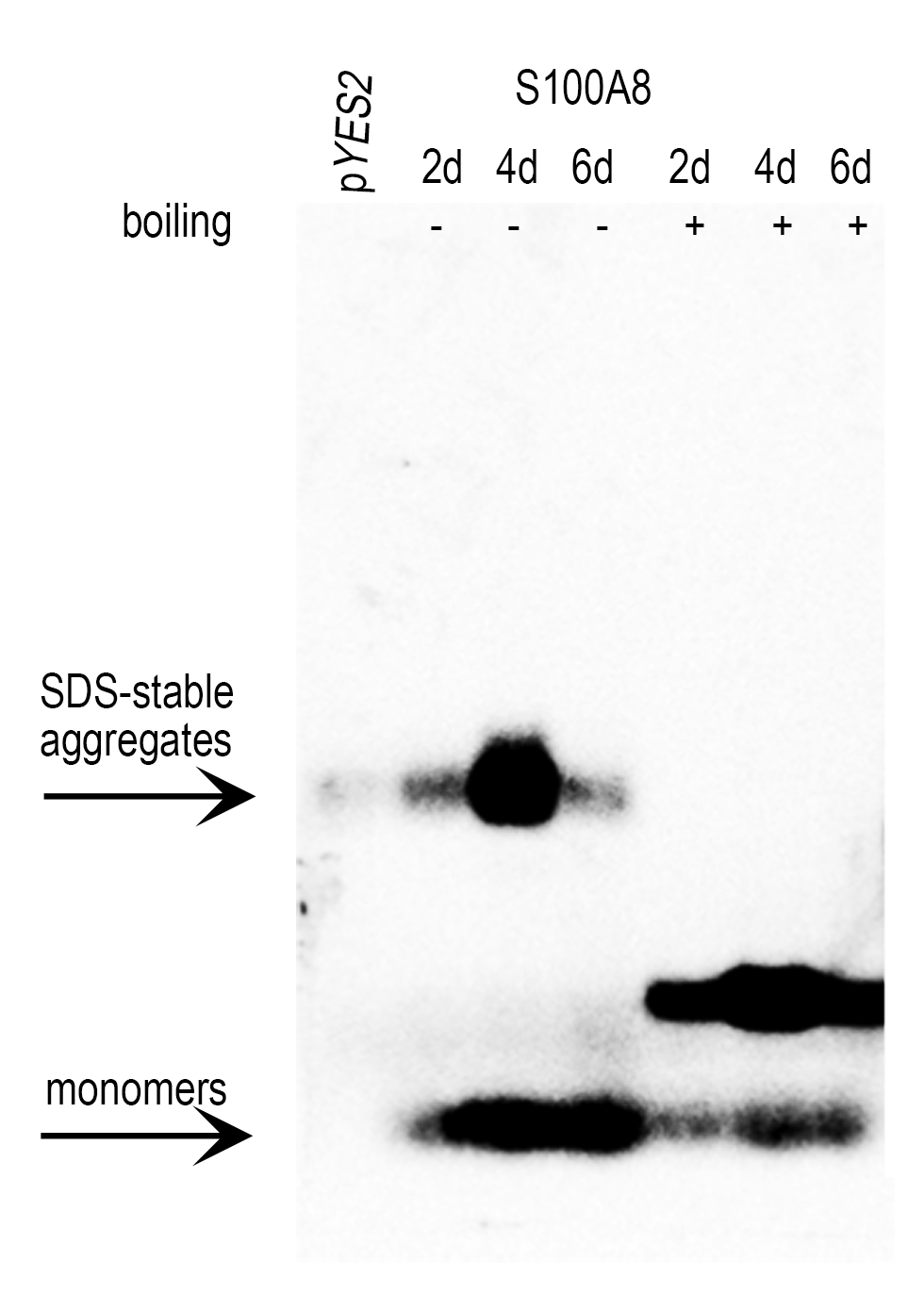

Supplement: Figure S1 — Analysis of S100A8 aggregates on SDD-AGE gels. Cell extracts were prepared from wild type cells that produced S100A8 protein after 2, 4, and 6 days of incubation on galactose plates. The yeast extracts were incubated for 10 min in 2% SDS sample buffer at room temperature with (+) or without (−) boiling and then loaded on the SDD-AGE gel and analyzed by Western blot using polyclonal anti-S100A8 antibodies. (TIF) [file pone.0058218.s001.tif]

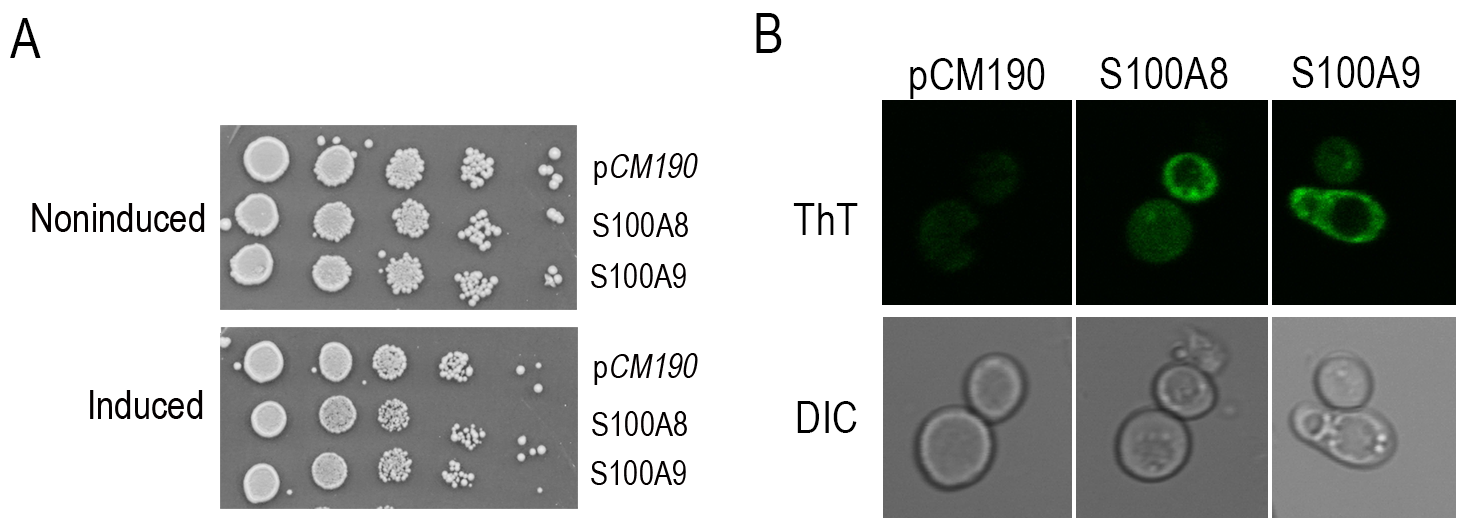

Supplement: Figure S2 — Aggregation and toxicity of S100A proteins induced by a TET on-off promoter system. (A) Ten-fold dilutions of wild type yeast cells transformed with pCM190 (empty vector), pTET-S100A8 or pTET-S100A9 were plated on glucose (inducing) or glucose with 5 µg/ml doxycycline (non-inducing) plates. (B) Spheroplasts of control and induced cells stained with ThT after 4 days of incubation on glucose plates. (TIF) [file pone.0058218.s002.tif]

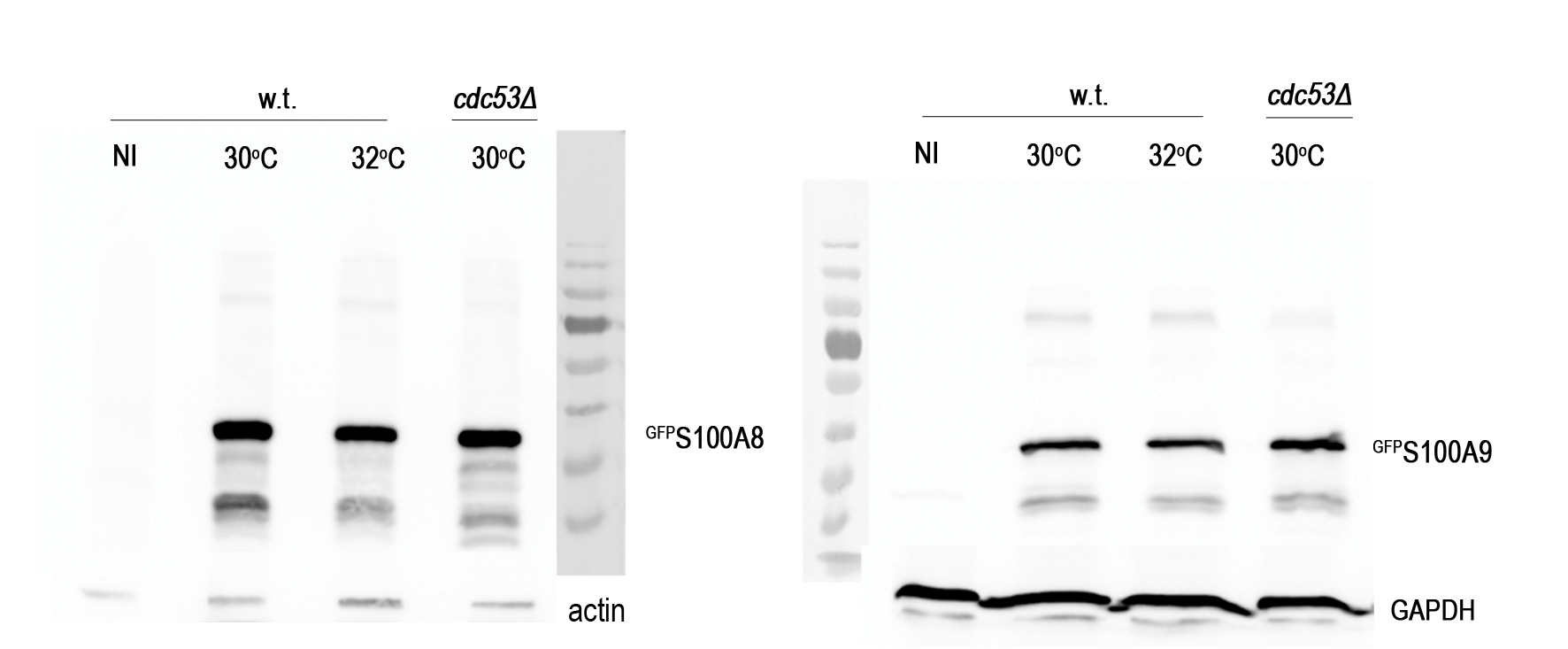

Supplement: Figure S3 — S100A8 and S100A9 protein levels in wild type and cdc53-1 cells. Wild type or cdc53-1 mutant cells expressing GFPS100A8 or GFPS100A9 were grown for 2 days on glucose or galactose plates at 30°C or 32°C. TCA precipitates of extracts were separated by 10% SDS-PAGE and analyzed by Western blot, using anti-actin or anti-GAPDH and anti-GFP antibodies. (TIF) [file pone.0058218.s003.tif]

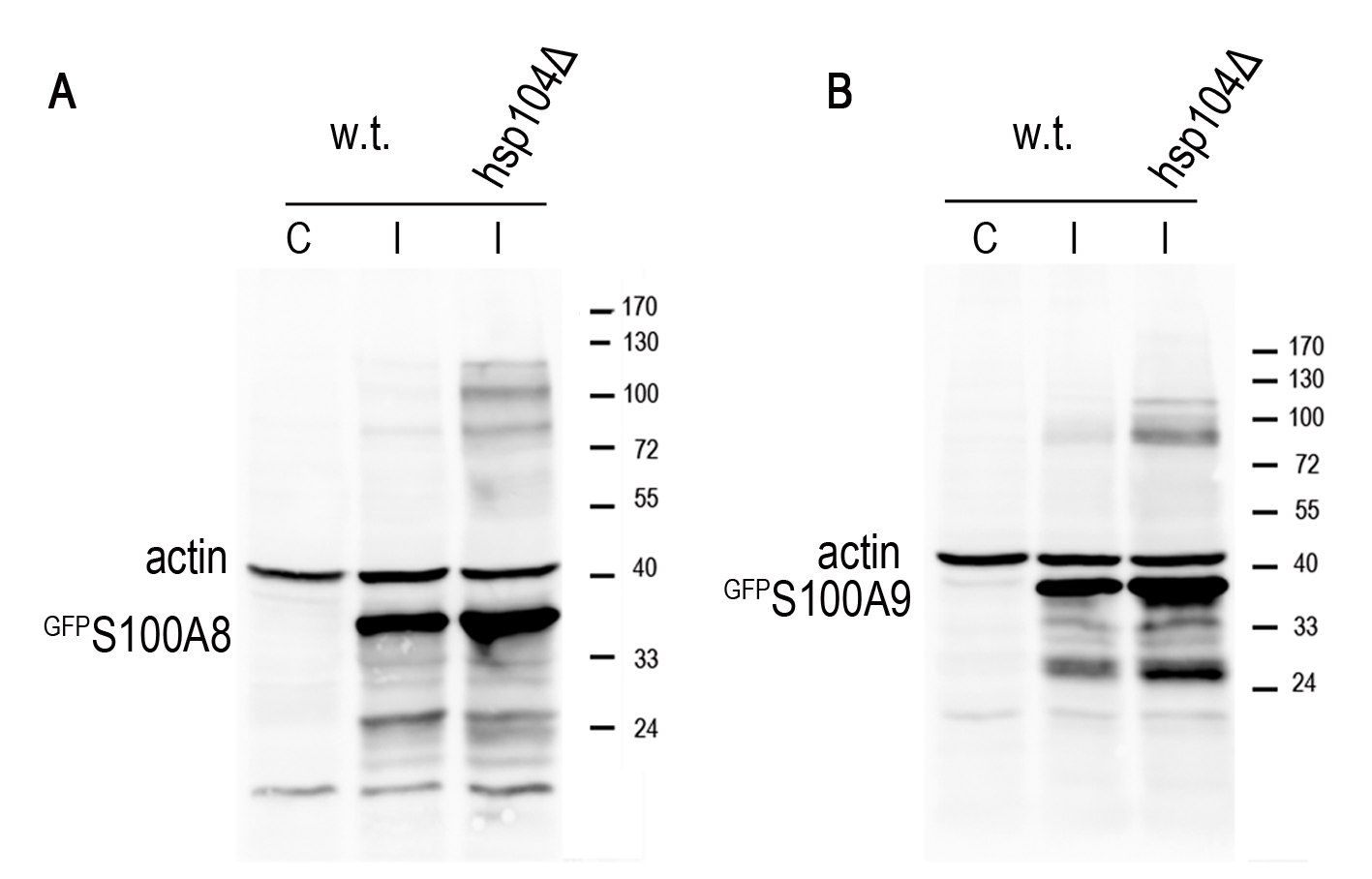

Supplement: Figure S4 — S100A8 and S100A9 protein levels in wild type and hsp104Δ cells. Wild type or hsp104Δ mutant cells expressing GFP-S100A8 or GFP-S100A9 were grown for 2 days on glucose or galactose plates. TCA precipitates of extracts were separated by 10% SDS-PAGE and analyzed by Western blot, using anti-actin and anti-GFP antibodies. (TIF) [file pone.0058218.s004.tif]
