# Supplementary material for: Aggregation of Human S100A8 and S100A9 Amyloidogenic Proteins Perturbs Proteostasis in a Yeast Model
Source: PLoS One. 2013 Mar 6;8(3):e58218. doi: 10.1371/journal.pone.0058218 (PMC3590125; doi:10.1371/journal.pone.0058218)
Supplement: Table S1 — Primer sequences. (DOC) [file pone.0058218.s005.doc]

**Table S1.** Primer sequences

| A8SalIF | GTCGACATGTTGACCGAGCTGGAG |
| --- | --- |
| A8Xho1R | CTCGAGCTACTCTTTGTGGCTTTC |
| mCherry EcoR1F | GAATTCATGGTGAGCAAGGGCGAGG |
| mCherrySalIR | GTCGACCTTGTACAGCTCGTCCATGCCGC |
| A9BamH1F | GGATCCATGACTTGCAAATGTCG |
| A9HinDIIIR | AAGCTTTTAGGGGGTGCCCTCCCC |
| S100A8HinD3F | AAGCTTATGTTGACCGAGCTGGAG |
| S100A8XhoR | CTCGAGCTACTCTTTGTGGCTTTC |
| S100A9BamH1F | GGATCCATGACTTGCAAATGTCG |
| S100A9Xba1R | TCTAGATTAGGGGGTGCCCTCCCC |
| A8BamH1F | GGATCCATGTTGACCGAGCTGGAG |
| A8HinDIIIR | AAGCTTCTCTTTGTGGCTTTCTTC |
