# Supplementary material for: Aggregation of Human S100A8 and S100A9 Amyloidogenic Proteins Perturbs Proteostasis in a Yeast Model
Source: PLoS One. 2013 Mar 6;8(3):e58218. doi: 10.1371/journal.pone.0058218 (PMC3590125; doi:10.1371/journal.pone.0058218)
Supplement: Table S2 — Plasmids for expression in yeast. (DOC) [file pone.0058218.s006.doc]

**Table S2.** Plasmids for expression in yeast

| **Plasmids for protein expression in yeast** | **Source** |
| --- | --- |
| p*YCPGAL-GFP* |  |
| p*GFP-S100A8* | This study |
| p*GFP-S100A9* | This study |
| p*mCherry-S100A8* | This study |
| p*YES2* | Invitrogen |
| p*YES2-S100A8* | This study |
| p*YES2-S100A9* | This study |
| p*103Q-GFP* |  |
| p*GAL-Sc104(WT)* |  |
| p*CM190* |  |
| p*TET-S100A8* | This study |
| p*TET-S100A9* | This study |
